# Beyond Aesthetics: Imaging-Based Evaluation of Carboxytherapy in Periorbital Hyperpigmentation

**DOI:** 10.3390/jcm15103776

**Published:** 2026-05-14

**Authors:** Rauf Hamid, Merve Nil Bayramoğlu, Sabri Şirolu, Osman Aykan Kargın, Seyfullah Halit Karagöz, Emrecan Sarı, Zekayi Kutlubay, Fatih Gülşen

**Affiliations:** 1 Department of Radiology, Republic of Türkiye Ministry of Health, Sungurlu State Hospital, 19300 Çorum, Türkiye; 2 Faculty of Medicine, Department of Dermatology and Venereology, Istanbul Medipol University, 34810 İstanbul, Türkiye; mervenilakyuz@hotmail.com; 3 Department of Radiology, Cerrahpaşa Faculty of Medicine, Istanbul University–Cerrahpaşa, 34098 Istanbul, Türkiye; sabri.sirolu@gmail.com (S.Ş.); sehekaragoz@gmail.com (S.H.K.); drfgulsen@yahoo.com (F.G.); 4 Department of Radiology, Istanbul Physical Therapy and Rehabilitation Training and Research Hospital, 34180 Istanbul, Türkiye; aykankargin@gmail.com; 5 Department of Radiology, Republic of Türkiye Ministry of Health, Çatalca Ilyas Çokay State Hospital, 34540 Istanbul, Türkiye; emrecan.sari.9165@gmail.com; 6 Department of Dermatology, Cerrahpaşa Faculty of Medicine, Istanbul University–Cerrahpaşa, 34098 Istanbul, Türkiye; zekayikutlubay@gmail.com

**Keywords:** periorbital hyperpigmentation, infraorbital dark circles, carboxytherapy, ultrasonography, superb microvascular imaging, shear-wave elastography

## Abstract

**Background:** In this study, we radiologically assessed potential increases in microvascularity, extracellular matrix-related changes, and tissue viscoelasticity following carboxytherapy for periorbital hyperpigmentation (POH). We also analyzed the correlation between radiological changes and clinical outcomes and explored implications for future outpatient selection, as well as the potential to predict treatment success based on radiological–clinical correlations. **Materials and Methods**: The present study included 78 patients (76 women and 2 men) aged over 18 years with Fitzpatrick skin types I–V and moderate-to-severe infraorbital dark circles who applied for treatment at the Dermatology Department in the Cosmetology Unit of Cerrahpaşa Medical Faculty Hospital. Each patient was given manual, pressure-controlled injections of sterile CO_2_ into the upper and lower eyelids for 7 weeks, with one round of treatment per week. We conducted dermatoclinical and radiological evaluations, including measurements of epidermis–dermis thickness and SWE, musculus orbicularis oculi pars pretarsalis thickness, and cSMI vascular index percentage, as well as SOOF tissue SWE (measured in kPa). These analyses were performed on both lower eyelids before treatment and at 1 month and 6 months after treatment. **Results**: After treatment, VAS scores improved significantly. Grayscale ultrasonography showed significant increases in epidermis–dermis and orbicularis oculi thickness at 1 and 6 months (*p* < 0.05). SMI presented a significant increase in vascular index at both follow-ups (*p* < 0.05). SOOF SWE values increased significantly at 1 and 6 months, whereas epidermis–dermis SWE did not. Procedural pain was common, and 25 participants withdrew during the 7-week period due to discomfort. Injection depth was not confirmed by real-time imaging, and adverse events were not graded using a standardized classification system. Therefore, tolerability and procedural safety should be interpreted with caution. **Conclusions**: Carboxytherapy was associated with improvements in clinical outcomes and radiological parameters among patients who were able to tolerate the procedure, including increased microvascularity on SMI and changes suggestive of extracellular matrix-related alterations. These improvements were maintained at the 6-month follow-up, indicating temporal persistence of the observed findings. However, due to the absence of a control group, the results should be interpreted with caution, and further randomized controlled studies are required to confirm these findings and establish causality.

## 1. Introduction

Periorbital hyperpigmentation (POH) is a common condition that accounts for a significant number of patients presenting with skin discoloration [1]. Patients present with semicircular, black-pigmented patches in the periorbital region. In some cases, these patches extend to the upper eyelids, eyebrows, and malar regions [2]. Reported rates of occurrence vary widely in the literature. Studies have reported that up to 78% of the general population may exhibit periorbital hyperpigmentation, with higher prevalence rates observed in Asian and African populations [3]. The mean age of onset is 32, with women aged 16–45 comprising most cases [4].

POH is histologically characterized by dermal melanin incontinence, increased amounts of melanosomes and vesicular melanin, perivascular lymphocyte infiltration, and scattered dermal melanophages [3].

POH has a multifactorial etiology. Contributing factors include UV exposure, inflammation, vascular changes, and lifestyle factors such as tobacco and alcohol use, sleep deprivation, and restricted fluid intake [5,6,7,8,9].

Anatomical factors may also contribute to the development of POH. These factors include a tear trough, a thinner lower eyelid with superficial vasculature, a lack of adipose tissue over the orbicularis oculi pars palpebralis, and skin thinning due to subcutaneous fat loss around the orbit [9,10].

Treatment of POH involves identifying and eliminating etiopathogenetic factors and, when appropriate, aesthetic modalities. Synthetic and naturally derived depigmenting active topical formulations are the primary therapeutic strategy [1]. In addition, hyaluronic acid injection is used for under-eye hollowness accompanying pigmentation [11].

Current and new treatments include laser therapy, autologous fat transplantation, platelet-rich plasma (PRP), and carboxytherapy, all of which are used alone or in combination with topical agents [12,13,14,15].

Recent randomized and comparative studies have reported clinical improvement with carboxytherapy, including comparisons with lasers, microneedling-based approaches, and platelet-rich plasma. However, outcome heterogeneity persists across studies, as reflected in differing efficacy estimates, assessment tools, and follow-up durations. A recent meta-analysis published in 2025 further emphasized this variability, highlighting limitations related to study design, treatment protocols, outcome measures, and patient characteristics, as well as the scarcity of objective imaging-based endpoints. Objective imaging correlates such as microvascularization, tissue stiffness, and layer thickness have been less frequently evaluated in POH carboxytherapy studies. Moreover, many existing studies lack controlled designs, limiting the strength of causal inferences.

The mechanism of carboxytherapy treatment involves the formation of an O_2_–CO_2_ imbalance under the skin following intradermal or subcutaneous CO_2_ injection. This imbalance has been reported to be associated with fibroblast proliferation, collagen deposition, and the release of vascular endothelial growth factor (VEGF). VEGF stimulates vasculogenesis and increases skin elasticity [8], leading to collagen deposition, lipolysis stimulation, and deposit destruction. For this reason, carboxytherapy is widely used in current aesthetic–clinical practice for skin lifting, rejuvenation, striae alba, and lipolysis regulation [9].

Carboxytherapy outcomes align with the variability of other treatment options; some studies report improvement, while others show inconsistent results [16,17,18]. Radiological imaging may help identify anatomical response modifiers and objectively measure outcomes.

This study aimed to assess radiological support for possible physiopathologic changes, examine the correlation between radiological and clinical changes, and determine the compatibility of radiological changes with clinical findings. The results were used to explore imaging correlates that may inform patient selection in future studies and treatment success based on radiologic–clinical correlation by evaluating thickness, elasticity, and microvascularization in the epidermis, dermis, orbicularis oculi, and suborbicularis oculi fat pad (SOOF) using superb microvascular imaging (SMI), shear-wave elastography (SWE), and conventional gray-scale ultrasonography.

## 2. Materials and Methods

This study was conducted in accordance with the Declaration of Helsinki. The hospital’s Ethical Committee approved this study.

We included 78 patients (76 females and 2 males) who presented to our dermatology department with moderate-to-severe infraorbital dark circles and were classified as having Fitzpatrick skin types I–V. The sample size was determined based on the available literature. Our exclusion criteria for this study were previous treatment of any kind for POH, chronic systemic disease, a past medical history of keloid scars, receiving immunosuppressants or PgF2a treatment, pregnancy, or lactation. Informed written consent was secured from all patients. In total, 53 patients completed the study, and 25 voluntarily withdrew during the 7-week treatment period due to discomfort. Baseline demographic and clinical characteristics of completers and non-completers were comparable based on available data (see Supplementary Table S1).

CO_2_ injections were administered subepidermally into the orbital skin in both periorbital regions using a Skymedic DIOXAGE^®^ device. The Skymedic DIOXAGE® device was sourced from Skymedic, Isaac Peral 91, 08224 Terrassa, Barcelona, Spain. In the injection protocol, a 32-gauge needle was used with a targeted injection depth of 6 mm. Although a nominal injection depth of approximately 6 mm was targeted based on device parameters, injections were performed in a controlled subepidermal/subcutaneous plane, and the effective depth likely varied according to individual periorbital anatomy. Real-time tissue expansion and resistance during CO_2_ delivery were used as procedural indicators to avoid unintended deep tissue penetration. Manual mode was selected, and sterile, warmed CO_2_ gas was injected at 50 mL/min under controlled pressure and flow. Although the injected volume varied according to each patient’s periorbital morphology, the mean volume was 50–70 mL per eye. The subepidermal injection of sterile carbon dioxide was terminated before the CO_2_ spread to the perizygomatic area. The procedure was conducted in two rounds, targeting two distinct points on the upper and lower lateral eyelids. When transitioning from the first to second pass, a mean waiting time of 10 min was allowed for the injected sterile CO_2_ gas to fully diffuse and the eyelid to return to its original form. The procedure was performed once per week for seven weeks. Patients were asked to use regular sun protection during the application period to avoid possible UVA-mediated melanosis.

Dermatological and radiological evaluations were performed for both eyes before treatment and at 1 and 6 months after treatment. We recorded Fitzpatrick skin types and POH pigmentation levels according to La Padula et al.; POH types, as described by Huang et al.; and smoking status, sleep, and hydration habits [19,20]. To clinically analyze hyperpigmentation, the primary outcome was the visual analog scale (VAS). Before the first session and 1 month after the last session, photographs were taken before and after carboxytherapy with a 12-megapixel camera under similar lighting conditions. The pre-procedure, one-month, and six-month post-carboxytherapy photographs were evaluated together, placed on a separate scoring sheet, and scored by 2 dermatologists. Patients were also asked to rate the photos using the VAS. Patients and doctors were asked to rate their overall satisfaction with the procedure on a scale of 1–5 (none/low/moderate/good/very good). Photographic evaluations were performed independently by two dermatologists in a blinded manner, using randomized image order and without access to patient identity or timepoint information. The observers evaluated the images separately, and their assessments were compared. In cases of discrepancy, a consensus was reached through joint review. Blinding was achieved by anonymizing all images and removing any identifying information, including patient identity and timepoint labels. Images were presented in a randomized order using a computer-generated sequence to ensure that evaluators were unaware of the treatment stage.

Although injections were administered to both upper and lower eyelids, radiological measurements were standardized to the lower eyelid. This region provides a more stable and reproducible ultrasonographic window, whereas the upper eyelid is more susceptible to variability due to the presence of the eyebrow, thinner tissue layers, and increased motion-related and probe-induced artifacts.

For the radiological evaluation, an 18 MHz linear transducer (Canon Medical Systems Corporation, Aplio i800 Platinum, Tokyo, Japan) was used for conventional ultrasonographic gray-scale, SMI, and SWE examinations to evaluate the tissue before and at 1 and 6 months after treatment. Ultrasonographic gray-scale, SMI, and SWE examinations were performed with the patients supine and their eyelids completely closed but not squeezed. A gel pad was applied to the eye socket, and a probe was placed perpendicular to the globe midline plane. In gray-scale ultrasound, we recorded the thickness of the orbicularis oculi pars pretarsalis from the midline, 1 cm lateral to the lens in the inferior tarsal area, and approximately 3 cm from the orbicularis retaining ligament, and the mean epidermis–dermis thickness. In SMI, the free-hand region of interest (ROI) was used to delineate the borders of the musculus orbicularis oculi pars pretarsalis (MOOPP). The static cSMI mode imaging parameters were low speed and high frame rate (PRF: 0.9–1.2 kHz, 42–49 frames/s [fps]). The vascular index (VI) of the manually drawn free-hand ROI was expressed as a percentage and evaluated semi-quantitatively according to Adler’s model [21]. All quantitative imaging measurements were obtained from the lower eyelid to ensure anatomical consistency and reduce measurement variability across time points.

**Figure 1:**
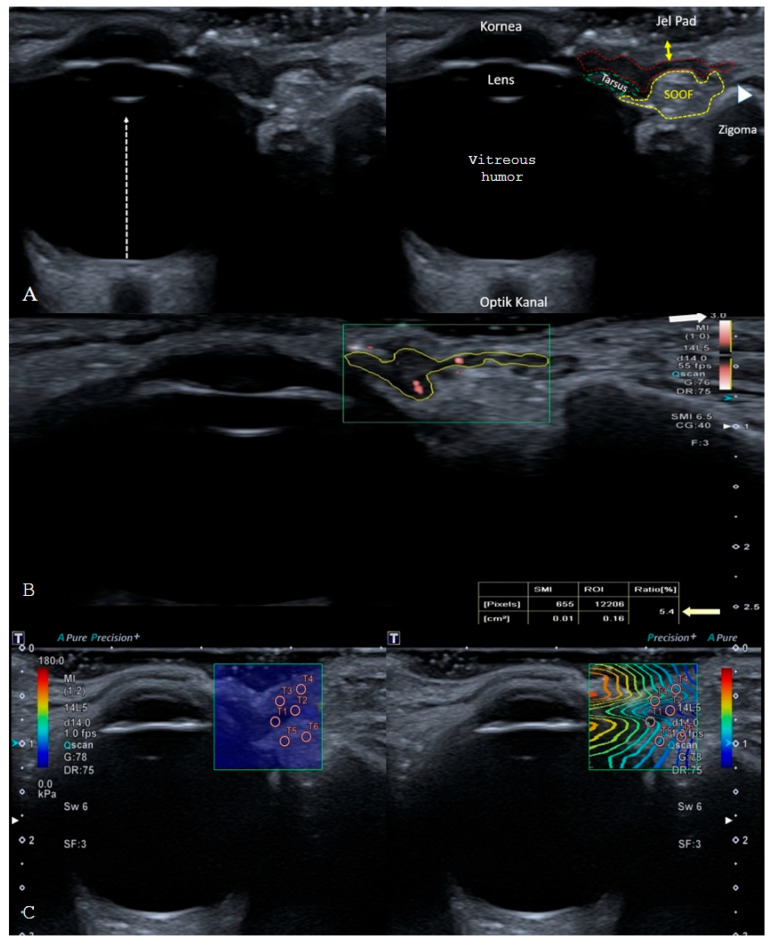
**(A**)**.** Sonoanatomy of the lower eyelid when the cornea, lens, and optic canal are aligned in the same plane. Epidermis–dermis (double-headed yellow arrow), orbicularis oculi muscle, pretarsal part (red outlined area), suborbital fat pad (yellow outlined area), tarsus (green outlined area), orbicularis retaining ligament (white arrowhead) (**B**)**.** The vascular index of the orbicularis oculi muscle, pretarsal portion, delineated using a free-hand region of interest (ROI), with a velocity scale limit of 3 cm/s (white arrow), and the SMI ratio (white arrow) (**C**)**.** Shear wave stiffness, expressed in kPa, was evaluated in the epidermis–dermis, orbicularis oculi muscle, and suborbicularis oculi fat (SOOF) using two ROIs for each tissue, each with a 2-mm diameter, covering a total assessed area of 1 cm^2^.

The 2D-SWE mode was used for the elastographic evaluation of the orbicularis oculi pars pretarsalis and SOOF. Single-shot mode (6 MHz frequency, 0.2 frames/s [fps]) was used to obtain quantifiable results. We measured the shear-wave stiffness of 2 ROIs of 2 mm in diameter in the epidermis–dermis, orbicularis oculi, and SOOF tissues in kPa (Figure 1). Radiological evaluations were performed in real time by two radiologists simultaneously on the same patient, allowing immediate comparison of measurements. Subsequently, all imaging data were independently reviewed by a third radiologist to ensure consistency and validation of the findings. Although complete blinding of radiological assessments was not feasible due to the real-time nature of ultrasonographic examinations, this multi-observer approach was implemented to reduce operator-dependent bias and enhance measurement reliability.

Data were presented as mean ± standard deviation for continuous variables or as frequencies (%) for categorical variables in the descriptive statistics of the results obtained pre-treatment and at 1 and 6 months post-treatment. The Kolmogorov–Smirnov test was used to assess the distribution of data. Most variables did not meet the assumptions of normal distribution; therefore, non-parametric tests were preferred throughout the analysis. For paired comparisons of pre- and post-treatment measurements, the Wilcoxon signed-rank test was used. For comparisons across three time points (baseline, 1 month, and 6 months), the Friedman test was applied. When appropriate, pairwise comparisons were performed using the Wilcoxon signed-rank test. Between-group comparisons for independent subgroups (e.g., sex, smoking status, sleep duration, and POH subtype) were performed using the Mann–Whitney U test. Categorical variables were compared using the chi-square test. A *p*-value < 0.05 was considered statistically significant, and SPSS 28.0 was used to analyze the data. Subgroup analyses (including smoking status, daily water intake, sleep duration, Fitzpatrick skin type, and POH subtype) were performed for exploratory purposes. No formal adjustment for multiple comparisons was applied, and these findings should be interpreted with caution.

## 3. Results

A total of 53 patients (51 females and 2 males) were included in this study. Overall, 50.9% of patients (*n* = 27) had Fitzpatrick skin type III, 34% (n = 18) had Fitzpatrick skin type II, and 15.1% (*n* = 8) had Fitzpatrick skin type IV. The degree of periorbital pigmentation was grade II in 77.4% (*n* = 46), grade I in 11.3% (*n* = 6), and grade III in 11.3% (*n* = 6) of patients. Pigmentation was a vascular type in 52.8% of patients, a mixed type in 37.7%, a structural type in 5.7%, and a pigmented type in 1.9%. Active smokers accounted for 35.8% of participants. The mean daily fluid intake was 1.8 L/day, and 75.5% of patients slept less than 7 h per day.

During the procedure, pain occurred in 92.5% of patients (*n* = 49), hematoma in 18.9% (*n* = 10), and swelling and edema in an area distant from the treated region in 15.1% (*n* = 8).

The patients’ pre-procedure VAS scores were 7.7 ± 1.3, whereas their post-procedure VAS scores were 4.6 ± 1.4. Physicians’ pre-procedure VAS scores were 7.7 ± 1.1, and their post-procedure VAS scores were 5.1 ± 1.3. Following the procedure, patient satisfaction with their treatment response was rated as good by 54.7%, moderate by 26.4%, and very good by 13.2%. Physicians’ satisfaction with the treatment response was rated as good by 50.9%, very good by 41.5%, and moderate by 5.7%. Patient- and physician-reported VAS scores decreased significantly after the procedure compared with the baseline (*p* < 0.05).

No significant differences were observed between female and male patient groups in pre- or post-procedure patient- or physician-reported VAS scores (*p* > 0.05). In both smoking and non-smoking groups, pre- to post-procedure decreases in patient and physician VAS scores were significant (*p* < 0.05). However, the magnitude of the VAS score reduction did not differ significantly between groups (*p* > 0.05). In the group with daily water consumption > 2 L, the post-procedure patient VAS score was significantly higher (*p* < 0.05), whereas the physician VAS score was lower. Sleep duration (≥7 h vs. <7 h) did not have a significant effect on pre- or post-procedure patient or physician VAS scores in either group (*p* > 0.05). Baseline patient VAS scores were similar across pigmentation-grade groups (*p* > 0.05). However, in all groups, significant reductions in both patient and physician VAS scores were observed after the procedure (*p* < 0.05). This reduction was more pronounced from both the patient’s and the physician’s perspectives among those with pigmentation grade II than among those with grade I (*p* < 0.05). However, there were no significant differences between pigmentation grade III and the other groups (*p* > 0.05). Baseline physician VAS scores were highest for the pigmentation grade III group (*p* < 0.05). After the procedure, this group also received higher physician VAS scores than did the other groups (*p* < 0.05) (Table 1).

In the Fitzpatrick skin type IV group, baseline patient and physician VAS scores were significantly higher than those in the Fitzpatrick skin type II and III groups (*p* < 0.05). In all skin types, post-procedure patient and physician VAS scores decreased significantly compared with those at baseline (*p* < 0.05). The magnitude of pre- and post-procedure reductions in patient and physician VAS scores did not differ significantly between groups (*p* > 0.05) (Table 2).

According to patient satisfaction rates, in both groups (none/low/moderate satisfaction and good/very good satisfaction), both patient and physician VAS scores decreased significantly after the procedure (*p* < 0.05). In the higher-satisfaction group, the post-procedure physician VAS score was lower, and the decrease in scores was greater (*p* < 0.05). However, the reduction in patient VAS scores did not differ significantly between groups (*p* > 0.05).

In patients with and without structural-type pigmentation, baseline patient VAS scores were similar (*p* > 0.05) and decreased significantly after the procedure in both groups (*p* < 0.05). However, the magnitude of reduction did not differ significantly between groups (*p* > 0.05). For physician VAS scores, a significant post-procedure reduction was observed in the non-structural group (*p* < 0.05), whereas no significant change was observed in the structural-type group (*p* > 0.05). Post-procedure physician VAS scores were higher in the structural-type group than in the non-structural group (*p* < 0.05). VAS scores for vascular, post-inflammatory pigmented, and mixed-type pigmentation decreased significantly from pretreatment levels, but there were no significant differences among these types of pigmentation.

The thicknesses observed on the grayscale ultrasounds of the epidermis–dermis and MOOPP both statistically significantly increased at 1 month and 6 months after treatment (*p* < 0.05) (Table 3). In addition to statistical significance, the absolute magnitude of change in radiological parameters was also considered. Although the observed differences were relatively modest in scale, they were consistent across time points (baseline, 1 month, and 6 months) and across multiple imaging modalities.

While the SWE values of the SOOF tissue statistically significantly increased at 1 month and 6 months after treatment, epidermis–dermis SWE values did not (Table 3). The lack of significant change in epidermis–dermis SWE values despite histological and thickness alterations may reflect technical limitations of shear-wave elastography in very low stiffness tissues. At low kPa ranges, SWE precision and reproducibility decrease, potentially masking subtle biomechanical changes.

**Figure 2:**
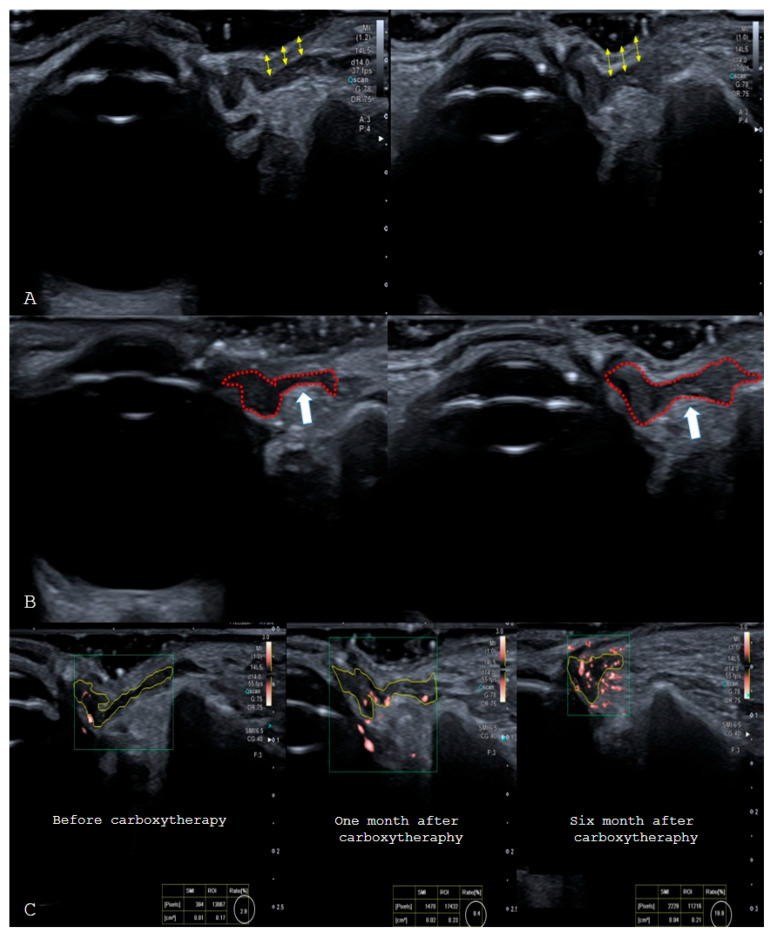
(**A**). At a measurement point 1 cm lateral to lower eyelid tarsal level, taken medial to the zygomatic eminence, an increase in epidermis-dermis thickness is demonstrated (double-headed yellow arrow) (**B**). At the same measurement point (1 cm lateral to lower eyelid tarsal level, medial to the zygomatic eminence), an increase in the thickness of the orbicularis oculi muscle (area within the red dotted outline) is demonstrated (level indicated by the white arrow) (**C**). At the same measurement point (1 cm lateral to the lower eyelid tarsal level, medial to the zygomatic eminence), an increase in the SMI vascular index percentage of the orbicularis oculi muscle is demonstrated before carboxytherapy, at the 1-month follow-up after carboxytherapy, and at the 6-month follow-up after carboxytherapy.

The vascular index value calculated with SMI for MOOPP was also statistically significantly increased at 1 month and 6 months after treatment (*p* < 0.05) (Table 3). All these values were also evaluated among patients stratified by POH subtype, gender, smoking status, sleep habits, and water consumption (Table 1). Additionally, patients were separated according to their satisfaction levels (no satisfaction, minor satisfaction, and moderate satisfaction were combined into one group, and good satisfaction and very good satisfaction were combined into a second group). Radiological values were compared between these groups (Figure 2).

## 4. Discussion

Periorbital hyperpigmentation is a cosmetic complaint that does not threaten an individual’s general health but adversely affects quality of life from a psychosocial perspective. Periorbital hyperpigmentation, characterized by brown-gray pigmented macules around both eyes, is influenced by personal habits, the anatomical structure of the region (muscle, fat, and bone tissue), and environmental factors. Topical agents, chemical peels, device-based therapies, and injection techniques can be used in management [22].

In a previous study, seven sessions of carboxytherapy were applied for POH in a cohort of 90 patients, and outcomes were analyzed 2 months after treatment. Both physicians and patients recorded preoperative and postoperative VAS scores, and a significant reduction in VAS scores was observed by both assessors after the procedure. A 50–60% reduction in periorbital pigmentation was also noted [23]. Another study examining 20 patients found that a four-week periorbital carboxytherapy protocol resulted in a statistically significant reduction in pigmentation in the periorbital area, with a complete response in 20% of patients. Additionally, the procedure was deemed effective and safe [17].

In another study evaluating the efficacy of platelet-rich plasma (PRP) and carboxytherapy in POH, a significant improvement in pigmentation was observed in both groups; however, treatment responses varied by technique and skin type [24]. In a study of 45 patients comparing the efficacy of carboxytherapy, vitamin C mesotherapy, and chemical peeling for POH, no significant differences in efficacy were observed between groups. Nevertheless, the authors argued that although the mesotherapy group experienced a higher rate of burning sensation as an adverse effect, the treatment’s efficacy was superior to that of carboxytherapy [25]. A previous study by Assaf et al. compared carboxytherapy with micro needling plus topical glutathione in POH. The authors noted that carboxytherapy was significantly superior in VAS scoring, patient satisfaction, and dark circle index [26]. A recent meta-analysis published in 2025 evaluated the available comparative evidence and concluded that both carboxytherapy and platelet-rich plasma are associated with clinically meaningful improvements in periorbital hyperpigmentation. However, no consistent superiority of either modality could be established due to substantial methodological and clinical heterogeneity across studies. Sources of heterogeneity included differences in treatment protocols, injection depth and volume; number of sessions; outcome assessment tools; follow-up duration; and variability in patient- and lesion-related characteristics, particularly the distribution of vascular, pigmentary, structural, and mixed periorbital hyperpigmentation subtypes. Collectively, these findings indicate that existing comparative data are insufficient to define definitive treatment hierarchies and emphasize the need for standardized protocols and objective outcome measures capable of delineating treatment-specific effects at the tissue level. In our study, both patient and physician VAS scores decreased significantly after the procedure compared with baseline. Subgroup analyses indicated that this reduction occurred independent of sex, smoking, sleep duration, Fitzpatrick skin type, vascular-type pigmentation, or structural-type pigmentation.

We compared patients according to the subtypes described by Huang et al. Decreases in patient VAS scores were similar in patients both with and without mixed-type lesions. The thickness of the MOOPP, however, was significantly higher in patients with vascular POH. In patients with mixed-type POH, the thickness of the MOOPP after carboxytherapy was significantly lower. These results suggest that the overall effects may be similar, but the proliferative effect of carboxytherapy is most prominent in patients with vascular POH and least effective in those with mixed-type POH.

The existing literature indicates that after CO_2_ application, perfusion, oxygenation, growth, and tissue regeneration increase due to a local myogenic mechanism resulting from the rightward shift in the Hb-O_2_ dissociation curve. Following local vasodilation in the arteriole and metarteriole, as well as in the precapillary sphincter, stimulation of VEGF-A and FGF-1 gene transcription in the tissue leads to increased vasculogenesis [27]. The SMI examination of the MOOPP indicated a statistically significant increase from the baseline VI at 1 and 6 months’ post-treatment. Notably, the 6-month post-treatment VI was significantly higher than the 1-month post-treatment VI. These findings may reflect sustained microvascular changes potentially consistent with angiogenic activity; however, direct evidence of angiogenesis cannot be established within the scope of this study. The increase in vascular index observed on SMI may indicate enhanced microvascularity, which is consistent with previously described angiogenic responses following CO_2_ therapy. Given the anatomical complexity of the periorbital region, injection depth and safety remain important considerations. Although no severe complications were observed in this cohort, the use of fixed nominal depth parameters without imaging guidance may introduce variability in tissue targeting. Incorporating ultrasound-guided approaches in future studies may help standardize injection planes and enhance both safety and reproducibility. The absence of severe complications in our cohort may reflect the use of a controlled injection technique and low flow parameters; however, this finding should be interpreted with caution, given the lack of imaging confirmation of injection depth and the absence of long-term follow-up. Although injections were performed in a controlled subepidermal/subcutaneous plane with continuous monitoring of tissue expansion, and no severe complications were observed, the exact injection depth and CO_2_ distribution were not confirmed by real-time imaging.

In the literature, increased local vascularization is associated with higher tissue temperature and extracellular matrix remodeling. These factors contribute to tissue regeneration through fibroblast proliferation and fibroblast-mediated collagen synthesis and accumulation, driven by the angiogenic effect and growth factor release in the tissue [28]. However, these mechanisms are derived from previously published histological and experimental studies and cannot be directly confirmed by the imaging-based findings of the present study. Nassar et al. reported that subepidermal CO_2_ injection increased matrix metalloproteinase-1 (MMP-1) expression, favoring collagen turnover, while overall collagen density increased [25]. These findings may be associated with changes in tissue composition that could contribute to the thickness of the lower eyelid dermis [28]. In line with the literature, our grayscale ultrasound examinations showed a statistically significant increase in the thickness of the lower eyelid epidermis and dermis at 1 and 6 months’ post-treatment. Once again, we observed that the thickness measurements at 6 months’ post-treatment were significantly higher than those at 1 month (Table 3). This finding may reflect the temporal persistence of the observed changes at 6 months; however, longer follow-up and controlled studies are required to confirm long-term effects.

Brochado et al. demonstrated that carboxytherapy stimulates cell proliferation through angiogenic and growth factors, potentially accompanied by nitric oxide-mediated vasodilator and antispasmodic effects [25]. To explore the hypothesis that this phenomenon could also promote myocyte proliferation, we measured the thickness of the MOOPP using gray-scale ultrasound.

The literature indicates that carboxytherapy promotes fibroblast proliferation, increases elastin synthesis, and enhances tissue regeneration through well-organized collagen fibers [28]. Biopsy results post-carboxytherapy have shown a significant increase in elastic fiber quantity [25]. Additionally, transcutaneous CO_2_ injection penetrates subepidermal white adipocytes, producing a lipolytic effect that stimulates adipolysis, as confirmed by histological analysis [13]. Epidermis–dermis and SOOF tissue SWE measurements were performed to evaluate this effect. Epidermis–dermis SWE measurements revealed no significant change, whereas SOOF SWE measurements indicated a significant increase at 1 month (Table 3). Since carbon dioxide injection targets a depth of approximately 6 mm, primary gas diffusion is thought to occur at approximately this depth. We hypothesize that this diffusion occurs due to the lack of an effect on the irregular, tight connective tissue in the dermis, which limits the impact of deeper carbon dioxide injection on the dermis’s viscoelasticity.

Even though MOOPP thickness increased less while VI increased more in men, we cannot hypothesize the reason for this phenomenon due to our small male patient sample size (Table 3).

In the literature, chronic smoking is associated with muscle dysfunction and volume loss, which is further associated with muscle apoptosis [29]. Before carboxytherapy, the thickness of MOOPP was statistically significantly lower in smokers. However, no statistically significant differences were observed between smokers and non-smokers in any post-treatment measurements (Table 3). This result indicates that smoking leads to a decrease in muscular volume. Nonetheless, a significant increase in volume was achieved after carboxytherapy.

The literature reports that increasing the water level in the tissue increases the thickness of collagen fibers, decreases the level of collagenase released by fibroblasts, and increases the tissue’s viscoelasticity by reducing the density and amount of collagen [30]. However, the effect of daily water consumption on carboxytherapy responses has not been previously analyzed in the literature. In our study, decreases in both patient and physician VAS scores were significantly greater in patients with daily water consumption < 2 L than in those with ≥2 L. In the group that consumed less than 2 L of water daily, lower eyelid SOOF elastography showed a low but statistically significant increase. In comparison, there was no significant difference in the group that consumed ≥2 L (Table 1). This finding suggests that hydration status may influence the response to carboxytherapy and indicates that further studies are needed to support these data.

The lower eyelid epidermis–dermis elastography values before carboxytherapy in the group with a sleep duration of 7–9 h were significantly lower than those in the group with a sleep duration of less than 7 h. However, there was no statistically significant change after carboxytherapy (Table 1). This result suggests that insufficient sleep contributes to tissue elasticity, while carboxytherapy has no statistically significant effect on epidermis–dermis elasticity.

The MOOPP VI in the group with a sleep duration of 7–9 h was significantly higher than that in the group with a sleep duration of less than 7 h. This result suggests that the vasodilator and long-term neoangiogenic effects of carbon dioxide were more pronounced in patients with adequate sleep duration.

It has been reported in the literature that insufficient and late sleep onset decreases hydration and thus the elasticity of the skin and subcutaneous soft tissue [31]. In the group with a sleep duration of less than 7 h, the SOOF elastography value on the first and sixth month after carboxytherapy showed a statistically significant increase compared to that in the pre-carboxytherapy group, while no statistically significant difference was found in the group with a sleep duration of 7–9 h (Table 1). This outcome suggests that the primary effect of carboxytherapy on white adipose tissue is likely related to lipolysis and elastin fiber synthesis, rather than tissue hydration.

In a study including 90 patients, the most common adverse events observed during seven sessions of carboxytherapy for periorbital pigmentation were ecchymosis, edema, and pain (25%); however, no adverse event leading to treatment discontinuation observed [23]. In an Iran-based cohort of 20 patients, erythema and edema were frequently observed during the procedure and regressed within 1 week with warm compresses and massage. Ocular twitching occurred in two patients, but no abnormality was detected on ophthalmologic consultation [17]. In our study, pressure and pain sensations occurred in 92.5% of patients during the procedure, while swelling and edema in an area distant from the treated region developed in 15.1%. Marked edema was observed the morning after the procedure and regressed within 24–48 h. During this period, if regression was observed, gentle periorbital massage and dispersion of subcutaneous gas were recommended. Mild-to-moderate hematoma persisting for 5–7 days after the procedure was observed in 18.9% of patients. While prior studies often report transient adverse effects without discontinuation, our real-world attrition during the 7-week protocol highlights tolerability as an important practical limitation and suggests that protocol parameters and patient counseling may influence adherence. Adverse events were recorded descriptively rather than graded using a standardized classification system (e.g., CTCAE), which limits detailed evaluation and comparability of safety outcomes across studies.

A headache lasting <24 h was recorded in 12% of patients. However, no patient discontinued treatment due to severe adverse effects. Although no severe adverse events were observed, the relatively high withdrawal rate due to procedural discomfort indicates that tolerability may be limited in a substantial proportion of patients. Therefore, the safety profile should not be interpreted solely on the basis of the absence of serious complications, and patient discomfort should be considered a clinically relevant limitation of the procedure.

In patients experiencing pain during the trial, the MOOPP SMI vascular index and SOOF elastography values increased significantly. In contrast, these values did not show significant changes in patients who did not experience pain (Table 3). Notably, the size of the potential space to be filled with gas may differ between patients, and some mechanical effects of this treatment may be evident. Therefore, in our practice, the amount of sterile gas injected into the subepidermal area during carbon dioxide injection was continued until tissue expansion, including fascial stretching, was achieved, and patient-reported discomfort occurred. However, optimal injection parameters, including volume and tolerability thresholds, should be further investigated in controlled studies. The significant thickening of the MOOPP suggests a potential effect on tissue structure; however, this interpretation remains speculative in the absence of direct histological validation. Although statistically significant changes were observed in radiological parameters, the clinical relevance of these absolute differences should be interpreted with caution. The magnitude of change in parameters such as epidermis–dermis thickness and SMI vascular index was relatively small, and in the absence of established minimal clinically important differences (MCIDs), it is not possible to determine whether these changes translate into clinically meaningful outcomes. While ultrasonographic techniques such as SMI and SWE are subject to measurement variability and operator dependency, the observed changes were consistent across multiple parameters, time points (baseline, 1 month, and 6 months), and independent observers. This consistency indicates that the detected differences are unlikely to fall within the range of measurement error alone and may reflect underlying tissue changes; however, this interpretation remains tentative and should be interpreted with caution. Overall, these interpretations should be considered hypothesis-generating, as the present study relies on imaging-derived surrogate markers without histological or molecular validation.

The limitations of this study include its single-center design, the small number of male participants, and the fact that carboxytherapy caused procedural pain in a large proportion of the patient population. Multicenter studies with larger populations and a more balanced sex distribution may provide a more statistically meaningful contribution to the literature.

The relatively high dropout rate (25 of 78 patients) may introduce attrition bias and affect the robustness and generalizability of the findings. The majority of discontinuations were related to procedure-related discomfort and appeared to occur without a specific temporal or clinical pattern. Although baseline demographic and clinical characteristics were comparable between patients who completed the study and those who withdrew, detailed comparative data are now presented in Supplementary Table S1 in the current manuscript. Therefore, this limitation should be considered when interpreting the results. Additionally, the analysis was conducted based on complete cases, as follow-up outcome data were not available for patients who withdrew early during the treatment period. This approach may further contribute to attrition bias and should be considered when interpreting the results. In addition, multiple subgroup analyses were conducted without adjustment for multiple comparisons, which increases the risk of type I error. Therefore, these results should be considered exploratory and interpreted with caution. Accordingly, the findings of the present study are primarily generalizable to patients who were able to tolerate repeated carboxytherapy sessions. The absence of follow-up data from patients who discontinued treatment may have led to an overestimation of treatment efficacy and an underestimation of tolerability-related concerns.

Another important limitation of this study is its single-arm, non-randomized design without a control group. The absence of a comparator group limits the ability to definitively attribute the observed improvements to carboxytherapy alone. Potential effects of natural variation, placebo response, observer bias, or external factors such as sun protection and lifestyle modifications cannot be excluded.

Furthermore, although the primary clinical outcome (VAS) is inherently subjective, we aimed to support our findings with objective imaging modalities, including SMI, SWE, and ultrasonographic measurements. Nevertheless, the lack of a control group remains a significant methodological limitation. Therefore, our findings should be interpreted with caution, and future randomized controlled studies are required to validate these results.

The use of VAS as a primary clinical outcome represents a subjective measure and may be influenced by observer- and patient-related biases. However, clinical evaluations were performed independently by two blinded observers using randomized and anonymized images, and interobserver agreement was assessed through comparison of evaluations, with discrepancies resolved by consensus, supporting the consistency of the findings. Radiological assessments were also conducted using a multi-observer approach, with two radiologists performing simultaneous real-time evaluations and a third radiologist independently reviewing all imaging data. Although complete blinding was not feasible due to the nature of ultrasonographic imaging, this methodology was intended to reduce operator-related bias and improve measurement reliability.

Another important limitation is the lack of real-time imaging guidance during the injection procedure. Therefore, the exact depth and anatomical distribution of CO_2_ delivery could not be objectively confirmed. Future studies incorporating ultrasound-guided injection techniques may provide more precise localization and further improve procedural safety assessment. Additionally, adverse events were not systematically graded using standardized criteria, which limits detailed evaluation of treatment tolerability.

## 5. Conclusions

This study provides a radiological view of the tissue changes expected following subepidermal sterile CO_2_ application, a current treatment for POH.

According to the study results, a statistically significant decrease in pigmentation was observed across all patient groups. The reduction in VAS scores was significantly greater in patients with daily water consumption < 2 L and those with grade II pigmentation. Sex, smoking status, sleep duration, and the presence of vascular pigmentation did not create significant differences in procedural outcomes.

Radiological imaging may serve as an objective adjunct to clinical assessment in patient selection and a follow-up to carboxytherapy for POH.

The observed radiological changes, including increases in SMI vascular index, tissue thickness, and elastographic parameters, were maintained at the 6-month follow-up. These findings may be associated with underlying tissue-level changes, including findings suggestive of extracellular matrix-related alterations and microvascular changes; however, they are based on imaging-derived surrogate markers and should not be interpreted as direct evidence of histological or molecular changes. Further randomized controlled studies are required to confirm causality and long-term efficacy, and to provide histopathological or molecular validation of these findings. In addition, the high treatment discontinuation rate due to procedural discomfort highlights the need for optimized treatment protocols and improved patient counseling and further suggests that patient selection and tolerability should be carefully considered when interpreting the clinical applicability of carboxytherapy.

Importantly, all proposed biological mechanisms, including increased microvascularity, extracellular matrix-related changes, and potential effects on tissue structure, are based on indirect imaging findings and should be considered hypothesis-generating. These mechanisms remain unverified and require confirmation through histological and molecular studies.

## Figures and Tables

**Table 1:** Patient-reported and physician-assessed Visual Analog Scale (VAS) scores before treatment and at 1 month after treatment according to age, sex, smoking status, daily water intake, and sleep duration. Values are presented as mean ± standard deviation. Statistical comparisons were performed between subgroups; a p value <0.05 was considered statistically significant.

	Age 18–49	Age 50–65	*p* Value	Woman	Man	*p* Value	Smo-ker	Non Smo-ker	*p* Value	Daily Water Intake < 2 L	Daily Water Intake > 2 L	*p* Value	Sleep Time < 7 h	Sleep Time > 7 h	*p* Value
Patient VAS score															
Before treatment	7.8 ± 1.2	7.5 ± 1.5	0.395	7.7 ± 1.3	7.3 ± 0.9	0.340	7.5 ± 1.3	7.8 ± 1.3	0.312	7.7 ± 1.3	7.6 ± 1.5	0.993	7.6 ± 1.3	8.1 ± 1.3	0.126
1 month after treatment	4.7 ± 1.5	4.6 ± 1	0.760	4.6 ± 1.5	4.8 ± 0.5	0.527	4.5 ± 1.1	4.7 ± 1.6	0.535	4.5 ± 1.4	5.3 ± 1.3	0.03	4.5 ± 1.2	5.1 ± 1.9	0.289
Doctor VAS score															
Before treatment	7.6 ± 1	7.8 ± 1.2	0.744	7.7 ± 1.1	7.8 ± 0.9	0.801	7.6 ± 0.9	7.7 ± 1.2	0.839	7.8 ± 1	7.3 ± 1.2	0.03	7.6 ± 1	7.8± 1.3	0.429
1 month after treatment	5.1 ± 1.3	5.1 ± 1.1	0.500	5.1 ± 1.3	5.3 ± 0.9	0.407	4.9 ± 1.2	5.1 ± 1.3	0.625	5 ± 1.2	5.3 ± 1.4	0.888	4.9 ± 1	5.6 ± 1.8	0.230

**Table 2:** Patient-reported and physician-assessed Visual Analog Scale (VAS) scores before treatment and at 1 month after treatment according to pigmentation degree and Fitzpatrick skin type. Values are presented as mean ± standard deviation. A p value <0.05 was considered statistically significant.

	Pigmentation Degree			*p* Value	Skin Type			*p* Value
	I	II	III		II	III	IV	
Patient VAS score								
Before treatment	7.8 ± 1.1	7.6 ± 1.2	8.6 ± 1.6	0.092	7.4 ± 1.3	7.7 ± 1.2	8.5 ± 1.2	0.048
1 month after treatment	5.5 ± 1	4.3 ± 1.1	6 ± 2.4	0.001	4.2 ± 1.4	4.8 ± 1.5	5.2 ± 0.9	0.011
Doctor VAS score								
Before treatment	7 ± 0.6	7.6 ± 0.9	9.2 ± 1.4	<0.001	7.3 ± 0.9	7.7 ± 1.1	8.3 ± 1.2	0.024
1 month after treatment	5.2 ± 1.1	4.8 ± 0.9	7 ± 1.8	<0.001	4.7 ± 0.9	5.3 ± 1.4	5.3 ± 1.5	0.146

**Table 3:** Ultrasonographic and elastographic measurements of the lower eyelid, orbicularis oculi muscle, and suborbicularis oculi fat (SOOF) at baseline, 1 month, and 6 months after treatment. Values are presented as mean ± standard deviation. *P* values represent pairwise comparisons between time points; a *p* value <0.05 was considered statistically significant.

Region/Structure	Parameter (Unit)	Baseline	1st Month	6th Month	*p* Value (Base-Line vs 1st Month)	*p* Value (Base-Line vs 6th Month)	*p* Value (1st Month vs 6th Month)
Lower eyelid	Epidermis-dermis thickness (mm)	1.1 ± 0.2	1.7 ± 0.3	2.0 ± 0.5	<0.001	<0.001	<0.001
	Epidermis-dermis elastography (kPa)	4.3 ± 1.1	4.3 ± 1.3	4.4 ± 2.0	0.740	0.880	0.937
Orbicularis oculi muscle	Pars pretarsalis thickness (mm)	0.9 ± 0.3	1.5 ± 0.4	1.9 ± 0.5	<0.001	<0.001	<0.001
	Pars pretarsalis SMI ratio (%)	3.3 ± 1.7	7.2 ± 3.2	9.2 ± 4.6	<0.001	<0.001	<0.001
SOOF	SOOF elastography (kPa)	5.0 ± 1.9	6.2 ± 2.8	6.9 ± 4.0	<0.001	<0.001	<0.001

## Data Availability

The original contributions presented in this study are included in the article/Supplementary Materials. Further inquiries can be directed to the corresponding authors.

## Supplementary Materials

The following supporting information can be downloaded at: https://www.mdpi.com/article/10.3390/jcm15103776/s1. Table S1. Baseline characteristics of completers and non-completers.
