# Supplementary material for: Beyond Aesthetics: Imaging-Based Evaluation of Carboxytherapy in Periorbital Hyperpigmentation
Source: J Clin Med. 2026 May 14;15(10):3776. doi: 10.3390/jcm15103776 (PMC13207061; doi:10.3390/jcm15103776)
Supplement: Supplementary file 1 [file jcm-15-03776-s001.zip › Supplementary_Table_S1.pdf]

Supplementary Table S1. Baseline characteristics of completers and non-completers

Variable	Completers (n=53)	Non-Completers (n=25)
Age (mean)	57	51
Sex (F/M)	49 / 4	25 / 0
Fitzpatrick type II (%)	18 (34.0%)	7 (28.0%)
Fitzpatrick type III (%)	27 (50.9%)	12 (48.0%)
Fitzpatrick type IV (%)	8 (15.1%)	6 (24.0%)
POH Grade I (%)	6 (11.3%)	4 (16.0%)
POH Grade II (%)	41 (77.4%)	19 (76.0%)
POH Grade III (%)	6 (11.3%)	2 (8.0%)
Smoking (%)	19 (35.8%)	12 (48.0%)
Sleep <7h (%)	40 (75.5%)	17 (68.0%)
